# Craniofacial Suture Maturation in Pycnodysostosis: A CBCT-Based Assessment of the Midpalatal, Zygomaticomaxillary Sutures and the Spheno-Occipital Synchondrosis

**DOI:** 10.1177/10556656261438345

**Published:** 2026-05-11

**Authors:** Cauby Maia Chaves Junior, Pedro Henrique Moreira Lima, Amanda Barbosa Pereira, José Luciano Pimenta Couto, Erlane Marques Ribeiro, Lucia Cevidanes, Fernanda Angelieri, Cristiane S. Fonteles

**Affiliations:** 1 Faculty of Pharmacy Dentistry and Nursing, Postgraduate Program in Dentistry, 204232 Federal University of Ceara (UFC), Fortaleza, Brazil; 2 Division of Pediatric Dentistry, School of Dentistry, Federal University of Ceara, Sobral Campus, Sobral, Brazil; 3 Division of Medical Genetics, Albert Sabin Children's Hospital, Fortaleza, Brazil; 4 Department of Orthodontics, UNC Adams School of Dentistry, 213004 University of North Carolina, Chapel Hill, NC, USA; 5 Department of Orthodontics and Pediatric Dentistry, School of Dentistry, 1259 The University of Michigan, Ann Arbor, MI, USA; 6 Department of Pediatric Dentistry, School of Dentistry, The University of Texas Health Science Center at Houston, Houston, TX, USA

**Keywords:** pycnodysostosis, craniosynostoses, sutures, craniofacial abnormalities, cone-beam computed tomography (CBCT), bone density

## Abstract

**Objective:**

This study aimed to evaluate the maturation stages of the midpalatal (MPS) and zygomaticomaxillary (ZMS) sutures, as well as the spheno-occipital synchondrosis (SOS), in patients with pycnodysostosis (PYCD) compared with controls, and to explore their associations with age and sex.

**Design:**

Cross-sectional study was matched by sex and age with a control group.

**Setting:**

In a secondary care center for patients with rare diseases in the state of Ceara (Brazil).

**Participants:**

18 participants (9 PYCD, 9 controls).

**Interventions:**

Sutures were analyzed using cone-beam computed tomography (CBCT) based on established classification systems.

**Main Outcome Measures:**

The stage of suture maturation was the main evaluation parameter. MPS and ZMS were evaluated for fusion stages, while SOS ossification was assessed using a 5-stage maturation scale. Statistical analyses included Pearson's chi-square, Fisher's exact tests, and Spearman's correlations to assess associations between suture stages and demographic or clinical variables.

**Results:**

Significant differences were observed between the groups in the maturation stages of the ZMS (*p* = .009) and MPS (*p* = .029), with earlier fusion in patients with PYCD, independent of age. SOS showed no significant differences between groups but exhibited a strong correlation with age (*p* = .001, *r* = .727). Associations with sex were observed only for the SOS (*p* < .001), indicating earlier fusion in females.

**Conclusions:**

Our findings confirm differences in the fusion stages of the MPS, and ZMS. However, further studies are needed to better understand the influence of sex and age on suture fusion, with age being a particularly significant parameter for the SOS.

## Introduction

Pycnodysostosis (PYCD) (OMIM 265800)^1^ is a rare autosomal recessive condition with a prevalence of 1.7 per million live births,^2,3^ linked to parental consanguinity in 30% of cases and distributed equally between sexes.^4^ The gene responsible for this condition is located on chromosome 1q21, encoding the lysosomal enzyme cathepsin K (CTSK), which is highly expressed in osteoclasts.^5,6^ This enzyme is responsible for degrading the organic bone matrix through resorption and remodeling mechanisms.^7^ Due to the reduced expression of CTSK in PYCD, patients often present with skeletal abnormalities resulting from increased bone density.^7–9^

PYCD also presents craniofacial alterations, such as brachycephaly, an obtuse mandibular angle, frontal and occipital prominence, midface hypoplasia, and open cranial sutures or fontanelles.^9,10^ Intraoral examinations may reveal anterior crossbite, posterior open bite, high-arched palate, tooth agenesis, severe dental crowding, prolonged retention of deciduous teeth, delayed eruption or impaction of permanent teeth, and poor oral hygiene associated with caries and periodontal disease.^9,11,12^

Although delayed or absent closure of maxillary sutures is a common feature of PYCD, some authors have reported craniosynostosis as a rare manifestation associated with this syndrome.^7,13^ Given the previously described phenotypic spectrum of craniofacial sutures in PYCD, an increasing interest has emerged in better understanding the patterns of facial suture consolidation. In addition, the mechanism by which CTSK dysfunction leads to craniosynostosis remains unclear. Because endochondral ossification and active bone turnover occur within the spheno-occipital synchondrosis, alterations in its maturation in PYCD could contribute to craniofacial discrepancies and the class III skeletal pattern frequently observed in these patients.

We therefore hypothesize that craniofacial growth sites—including the midpalatal suture (MPS), zygomaticomaxillary suture (ZMS), and spheno-occipital synchondrosis (SOS) exhibit distinct maturation patterns in PYCD. The aim of this study was to assess these craniofacial growth sites in individuals with PYCD, compare them with controls, and investigate potential associations between suture consolidation stages and variables such as sex and age. Facial suture and synchondrosis maturation were evaluated using a validated CBCT-based classification method introduced in the past decade, designed to stage facial suture maturation and examine its correlation with chronological and skeletal age.^14,15^

## Material and Methods

### Study Population

This was a clinical, observational, cross-sectional study approved by the Human Research Ethics Committee of the Federal University of Ceara (UFC, Fortaleza, Brazil protocol no. 1.063.126). All participants provided written informed consent prior to inclusion in the study and CBCT image acquisition. The sample comprised 9 participants diagnosed with PYCD, enrolled and monitored through the Outreach Dental Service for Infants and Children with Special Needs (SEMENTE Project) of the Postgraduate Program in Dentistry at UFC. The control group included 9 non-syndromic participants with Angle Class I occlusion, matched by age and sex, selected from a database of 150 CBCT scans obtained at a reference radiology clinic in Fortaleza, Ceara (Brazil). All data were anonymized, and the study adhered to the principles of the Declaration of Helsinki.

### Sample Size

Sample size and power calculations were performed using OpenEpi (version 3.01), an open source, web-based statistical tool (https://www.openepi.com/SampleSize/SSMean.htm). For this calculation, PYCD was considered an ultrarare autosomal recessive skeletal dysplasia, with fewer than 200 molecularly confirmed cases reported globally and an estimated prevalence of 1–1.7 per 1,000,000 individuals, as described in GeneReviews and OMIM.^16,17^ According to Moreira et al. (2019),^18^ who carried out a review of articles published between 1976 and 2018, Brazil and India report the highest number of documented cases, with 76.9% of Brazilian cases occurring in the Northeast region. Using a 2-sided Student's t-test to compare groups (PYCD versus controls), a significance level of α = 0.05 and power of 80% (β=0.20) yielded a minimum required sample size of 18 participants (PYCD, n = 9; controls, n = 9). Given the global rarity of this condition, the inclusion of 9 individuals with PYCD from a single center in a high-prevalence region represents a meaningful and scientifically appropriate sample size for clinical investigation.

### Image Acquisition

Cone-beam computed tomography (CBCT) images were obtained using an i-CAT^®^ scanner (Imaging Sciences, Hatfield, PA, USA) under the following exposure parameters: 120 kVp, 14 mA, and a field view of 23 × 17 cm and the scan time ranged from 20 to 40 s, with spatial resolution between 0.25 and 0.30 mm. DICOM files from each scan were exported and transferred to a desktop computer equipped with the InVivo 5 software for DICOM file visualization (Anatomage, San Jose, CA, USA).

### Image Analysis Procedures

The head orientation was verified or corrected, according to the natural head position in the sagittal, coronal, and axial planes. The cursor (the position indicator) of the image analysis software was positioned at the midsagittal plane of the patient in both coronal and axial views.

For MPS analysis,^14^ in the sagittal view, the patient's head was adjusted so that the anteroposterior long axis of the palate was horizontal. The software's horizontal orange line was placed along the palate and the most central cross-sectional slice in the superior–inferior dimension (i.e., from the nasal to the oral surface) was selected in the axial view and it was utilized for classification of the maturational stage of the midpalatal suture.

The ZMS analysis was performed according to the method described by Angelieri et al.^15^ In sagittal view, the horizontal cursor (orange line) was placed at the tip of the nose parallel to the palatal plane. At this inferior–superior cross-section determined in the sagittal view, the axial view then displayed a portion of the oblique ZMSs bilaterally. The anteroposterior cursor (purple line) then was positioned transversely through the ZMSs bilaterally. These procedures allowed visualization of the ZMSs also in the coronal view. In this coronal view, the vertical cursor (green line) first was positioned along the ZMS in one side. These orientation steps allowed visualization of the suture in all views (sagittal, axial, and coronal). For the interpretation of the maturational stage of the inferior portion of the ZMS, it was required to rotate the patient's head in a counterclockwise direction in the coronal view until the inferior portion of the ZMS was visualized appropriately in the sagittal view for the left side, and in the clockwise direction, for right side. Radiographic interpretation was performed in the cross-sectional slice that best allowed visualization of the long axis of the ZMS in each side (right and left) at the infraorbital (superior) and infrazygomatic (inferior) portions of the suture.

The SOS analysis was performed according to the method described by Bassed et al.^19^ At the midsagittal plane of the patient, selected in both coronal and axial views, the cross-sectional slice of the cranial base was evaluated in the sagittal view.

Due to the distinct phenotypic features of patients with PYCD, image acquisition of MPS, ZMS, and SOS slices was not blinded. However, the evaluation of these slices was performed at a different time, in a blinded manner, with no identification of the patient, group, or age.

### Calibration

For calibration purposes, 30 cervical vertebrae images and 30 midpalatal suture images from the same individuals were randomly selected from the total sample and reclassified by the same examiners after a 1-month interval. A weighted kappa coefficient was calculated to assess intraexaminer agreement for the CVM method and MPS maturation classification. Weighted kappa coefficients for intra-examiner agreement was 0.978 for the CVM method and 0.935 for MPS classification, indicating very high intra-examiner reproducibility. Twenty ZMS images were randomly selected from the total sample and reclassified by the same examiner 1 month later, with 100% intra-examiner reproducibility observed.

### Imaging Evaluation

All slices were organized by a single investigator into a PowerPoint presentation (Microsoft, Redmond, WA) with a black background, sequentially displayed on a high-definition monitor without any adjustments to contrast or brightness. CBCT slices were selected and blindly evaluated in the 3 spatial planes for the MPS, ZMS, and SOS by a single examiner specializing in this field.

The MPS was assessed using transverse axial slices of the palate, following the 5-stage system (A–E) previously described by Angelieri et al. (2013)^14^ (Figure 1): stage A (a relatively straight, high-density line is observed), stage B (a high-density jagged line with areas of 2 parallel lines separated by low-density spaces is observed), stage C (2 closely spaced, parallel, high-density lines separated by small low-density spaces are observed), stage D (2 jagged high-density lines in the maxillary portion of the palate indicate suture fusion in this region, with higher bone density in the palatine bone compared to the maxilla) and stage E (complete MPS fusion is observed, with parasutural bone density similar to that of other regions of the palate).

**Figure 1.**
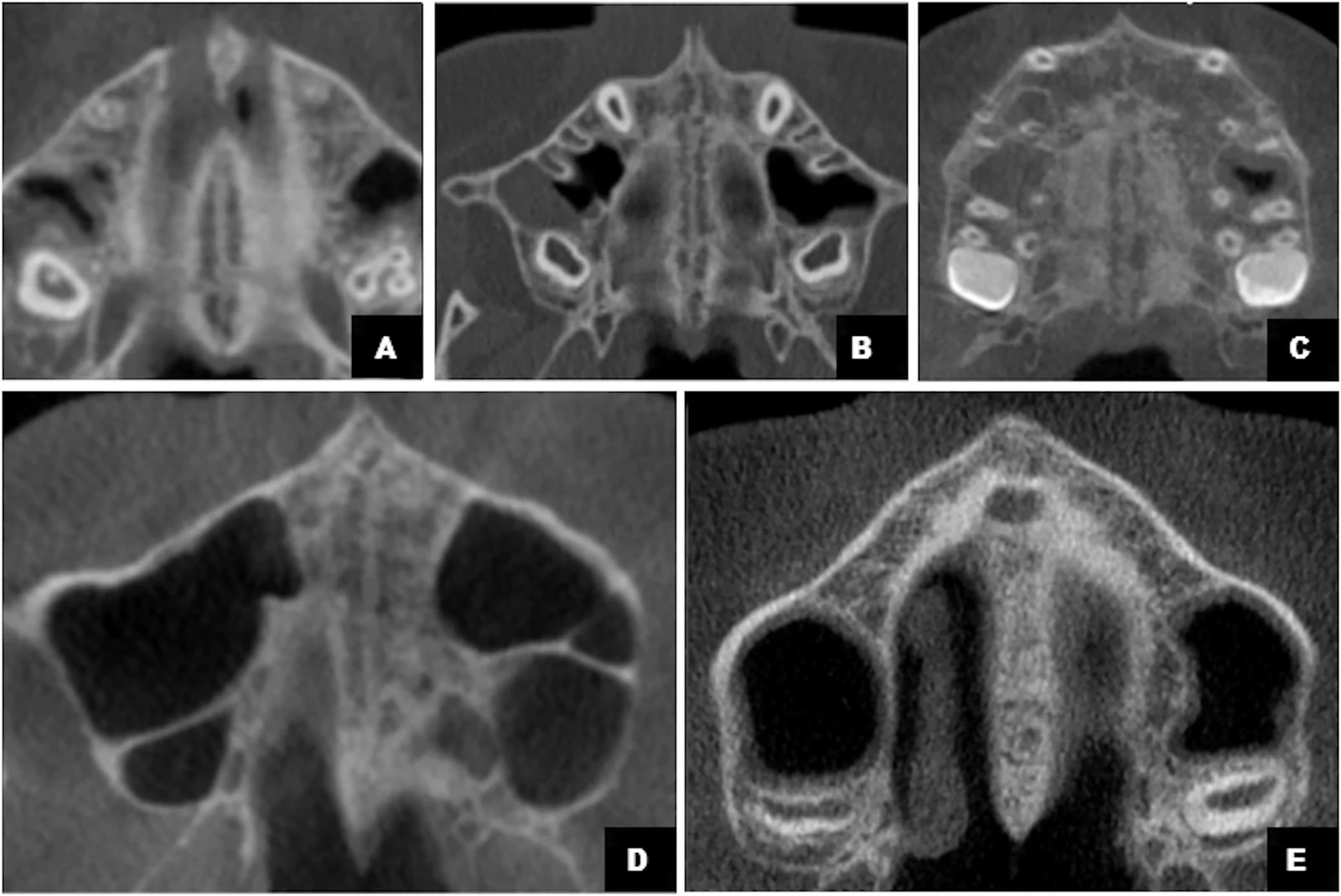
CBCT-based staging of midpalatal suture (MPS) maturation. Representative axial CBCT slices illustrating the 5 maturation stages (A–E) of the midpalatal suture proposed by Angelieri et al. (2013). Axial palatal slices were used for staging. The images demonstrate progressive morphological changes of the suture from an open configuration to complete fusion.

The ZMS was evaluated in its superior (infraorbital) and inferior (infrazygomatic) portions using sagittal slices to determine maturation, based on the 5-stage system (A–E) described by Angelieri et al. (2017)^15^ (Figure 2). Radiographic analysis was performed using sagittal slices (which allowed optimal visualization of the suture's long axis) at the infraorbital (superior) and infrazygomatic (inferior) portions of the suture on both sides. In stage A (a uniform, dense line with little or no interdigitation is observed. Parasutural bone appears less dense), in stage B (a thicker, jagged, high-density line with slight interdigitation is observed. There may be areas with 2 closely spaced, thin, parallel lines separated by low-density spaces), in stage C (2 thin, parallel lines separated by low-density spaces are observed), in stage D (partial suture fusion, usually in its inferior portion, with increased bone density in the area is observed) and in stage E (extensive fusion prevents suture visualization, with increased parasutural bone density).

**Figure 2.**
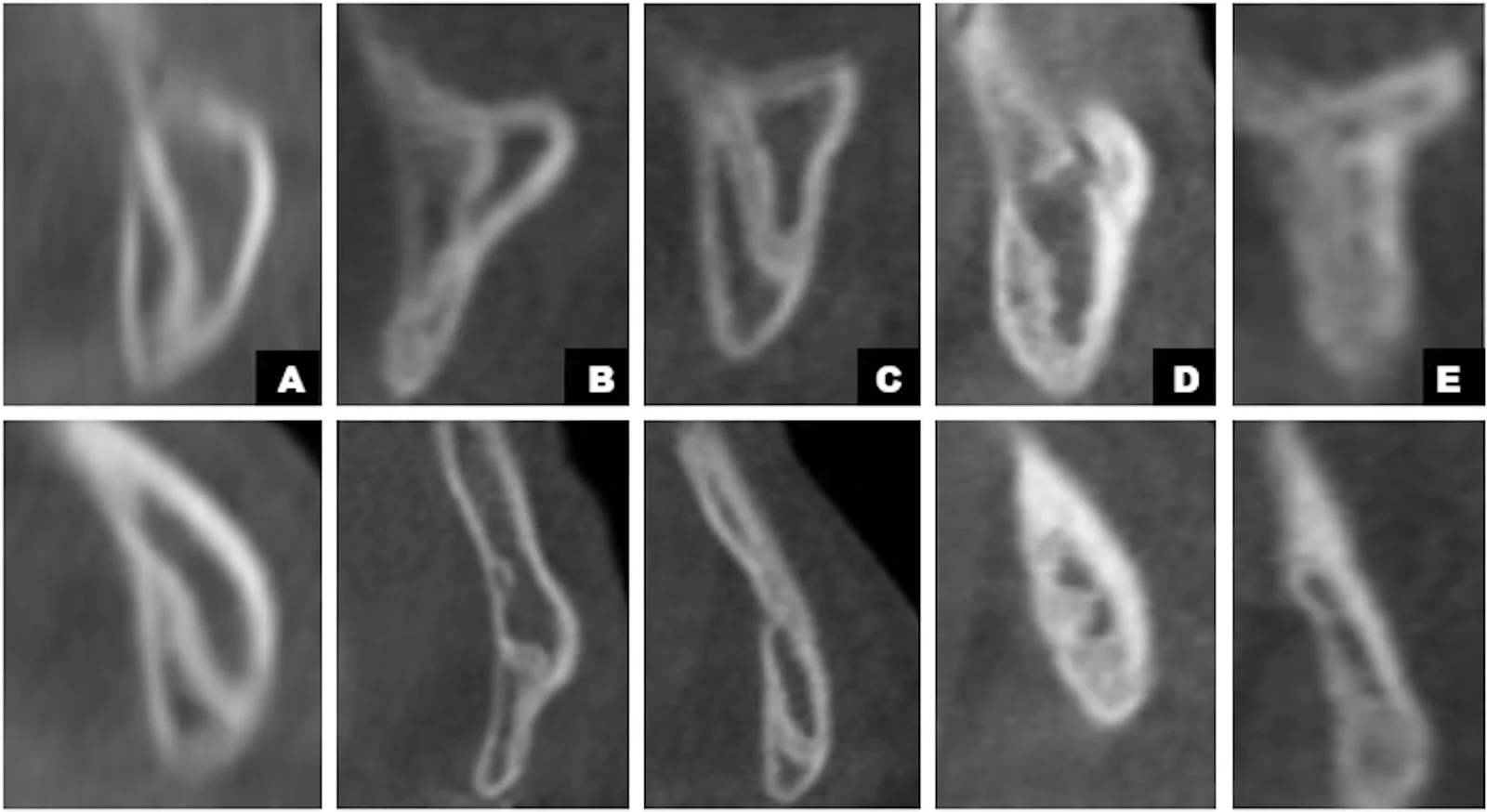
CBCT-based staging of zygomaticomaxillary suture (ZMS) maturation. Representative sagittal CBCT slices illustrate the 5 maturation stages (A–E) of the ZMS according to Angelieri et al. (2017). Sagittal views were used to visualize the long axis of the suture. Images correspond to the infraorbital (superior) and infrazygomatic (inferior) portions, demonstrating the progression from a clearly visible suture line to extensive fusion.

The ossification stage of the SOS was assessed using the 5-stage system (1–5) developed by Bassed et al. (2010)^19^ (Figure 3), through median sagittal slices of the cranial base. In stage 1 (the synchondrosis is completely open and unfused), in stage 2 (the superior border is fused, while the rest of the fusion site remains open), in stage 3 (half of the synchondrosis length is closed), in stage 4 (closure is nearly complete, but the site remains visible as a fusion scar) and in stage 5 (the site is entirely obliterated, resembling normal bone across its length).

**Figure 3.**
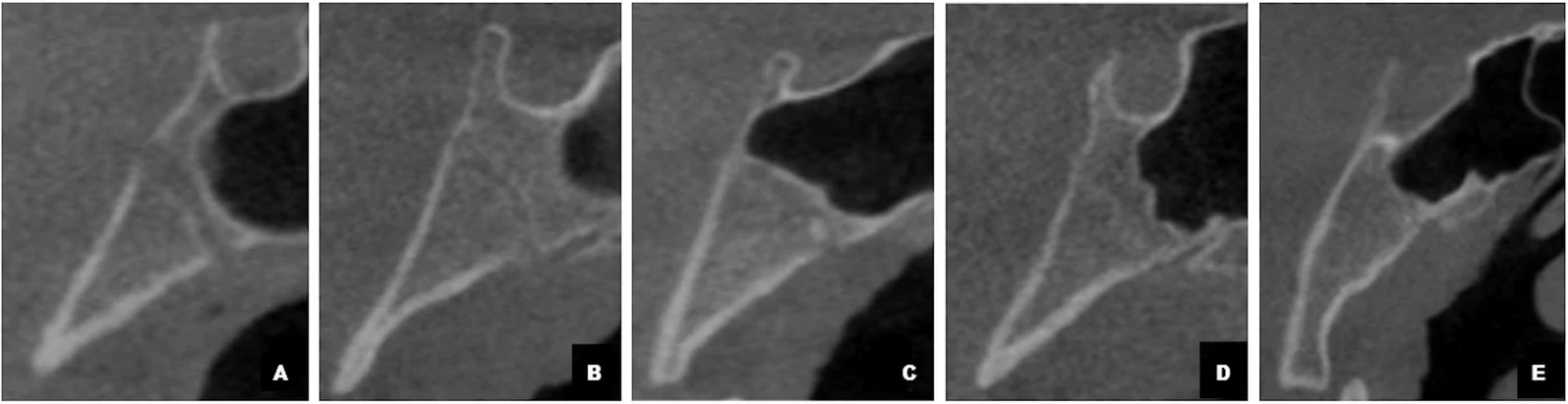
CBCT-based staging of spheno-occipital synchondrosis (SOS) ossification. Median sagittal CBCT slices illustrating the 5 ossification stages (1–5) of the SOS according to Bassed et al. (2010), demonstrating the transition from a completely open synchondrosis to complete obliteration. Staging was performed using midsagittal slices of the cranial base.

### Statistical Analysis

Data were tabulated in Microsoft Excel and analyzed using SPSS^®^ software version 20.0 with a 95% confidence interval. The MPS, ZMS, and SOS were classified alphanumerically and progressively coded on a 1–5 scale for suture closure level, analyzed as ordinal or ranked variables, and nonparametric tests were applied. Comparisons between groups (PYCD and control) were made using the Mann–Whitney U test.

Fisher's exact test was used to assess associations between suture consolidation stages and categorical variables such as sex and group. It was also applied when sutures were reclassified and grouped by the stage of closure as CLOSED versus NOT CLOSED. Results were presented as crude association coefficients using phi (φ) or Cramér's V (V^2^) with 95% confidence intervals (CIs) and *p*-values provided for reference (*p* ≤ .05). Finally, Spearman's rank correlation coefficients were calculated to assess relationships between suture classification and age.

## Results

### Sample Characteristics

The final sample comprised 18 participants, including 9 individuals with PYCD and 9 age- and sex-matched controls (7 females and 2 males). The PYCD group had a mean age of 16.99 ± 8.23 years (range: 3.25–30.41), while the control group had a mean age of 17.11 ± 7.55 years (range: 6.66–32.08). All participants in the PYCD group exhibited Angle Class III malocclusion, whereas all controls presented with Angle Class I. Within the PYCD group, 5 patients presented with an anterior crossbite, 1 with an edge-to-edge bite, and 3 with no overjet alteration. None of the control participants exhibited anterior crossbite.

### Suture and Correlation Analyses

In the evaluation of the MPS and ZMS sutures, all patients with PYCD demonstrated complete fusion, irrespective of chronological age. In contrast, within the SOS, only 2 patients with PYCD did not present with complete fusion. Participants in the control group exhibited fusion patterns consistent with their chronological age (Table 1).

**Table 1.** Distribution of Anthropometric Variables, Orthodontic Occlusion Parameters and 3-Dimensional Assessment of Sutures and Synchondrosis in the Pycnodysostosis and Control Groups.

	Anthropometric Data				Orthodontic Parameters				Consolidation of Sutures					
Variable	Age		Sex		Anterior crossbite		Angle Classification		MPS		ZMS		SOS	
Patients	PYCD	Control	PYCD	Control	PYCD	Control	PYCD	Control	PYCD	Control	PYCD	Control	PYCD	Control
01	17.16	16.08	F	F	YES	NO	3	1	E	D	E/E	D/D	5	5
02	9.58	10.66	M	M	NO	NO	3	1	E	B	E/E	C/D	2	2
03	3.25	6.66	M	M	NO	NO	3	1	E	B	E/E	C/C	1	1
04	30.41	32.08	F	F	NO	NO	3	1	E	E	E/E	E/E	5	5
05	13.66	13.3	F	F	YES	NO	3	1	E	C	E/E	C/C	5	5
06	25.25	20.16	F	F	YES	NO	3	1	E	E	E/E	E/E	5	5
07	19.08	18.3	F	F	YES	NO	3	1	E	E	E/E	D/D	5	5
08	21.5	23.25	F	F	ON TOP	NO	3	1	E	E	E/E	E/E	5	5
09	13.08	12.83	F	F	YES	NO	3	1	E	C	E/E	C/C	5	5

*Note*: PYCD, pycnodysostosis; MPS, midpalatal suture; ZMS, zygomaticomaxillary suture; SOS, spheno-occipital synchondrosis; F, female; M, male.

When analyzing participants collectively, age was positively correlated with the right ZMS (rho=0.478, *p = .*045), MPS (rho=0.522, *p = .*026), and SOS (rho=0.727, *p = .*001). When the groups were analyzed separately, a significant correlation with age persisted only for the SOS (rho=0.730, *p = .*025), in both the control and PYCD groups.

### Suture Consolidation and Its Association with Group and Sex

A comparison between groups revealed significant differences in the consolidation stages of the right ZMS (*p = .*009), left ZMS (*p = .*009), and MPS (*p = .*029). However, no significant difference was observed for SOS (p = 1.000) (Table 2).

**Table 2.** Comparison of Stages of Suture Closure Between Groups, PYCD and Control (N = 18).

Suture/synchondrosis	50th (Median)	*p*
Zygomaticomaxillary R	5 (3-5)	.009*
Zygomaticomaxillary L	5 (3-5)	.009*
Midpalatal suture	5 (2-5)	.029*
Spheno-occipital synchondrosis	5 (1-5)	1.000

*Note*: Mann–Whitney *U*-test. *p < .05

When data were analyzed by group, significant associations were observed between the consolidation stages of MPS (*p = .*029), right ZMS (*p = .*009) and left ZMS (*p = .*009) and the presence of PYCD, as determined by Pearson's chi-square test (Table 3). A significant association was also observed between SOS consolidation and sex (*p < .*001) (Table 4).

**Table 3.** Association Between the Stages of Suture Closure and the Variable Group (PYCD and Control).

Variables		Group (N = 18)					
Suture/synchondrosis	Stage^a^	Frequency^b^			*P*	V^2^	IC
		PYCD^c^	Control	Total			
Zygomaticomaxillary R	STAGE C	0 (2.0)	4 (2.0)	4	.009*	.707	.443-1.000
	STAGE D	0 (1.0)	2 (1.0)	2			
	STAGE E	9 (6.0)	3 (6.0)	12			
Zygomaticomaxillary L	STAGE C	0 (1.5)	3 (1.5)	3	.009*	.707	.443-1.000
	STAGE D	0 (1.5)	3 (1.5)	3			
	STAGE E	9 (6.0)	3 (6.0)	12			
Midpalatal suture	STAGE B	0 (1.0)	2 (1.0)	2	.029*	.620	.331-.892
	STAGE C	0 (1.0)	2 (1.0)	2			
	STAGE D	0 (0.5)	1 (0.5)	1			
	STAGE E	9 (6.5)	4 (6.5)	13			
Spheno-occipital synchondrosis	STAGE 1	1 (1.0)	1 (1.0)	2	1.000	.000	.038-.555
	STAGE 2	1 (1.0)	1 (1.0)	2			
	STAGE 5	7 (7.0)	7 (7.0)	14			

*Note*: Fisher's exact test. *p < .05.

a Zygomaticomaxillary R/L: Stage C: 2 thin lines separated by low-density spaces; Stage D: Partial suture fusion; Stage E: Multiple areas of fusion with increased parasutural bone density. Midpalatal suture stage: Stage B: A jagged, high-density line with areas showing 2 parallel lines; Stage C: 2 closely positioned, parallel, high-density lines; Stage D: 2 jagged, high-density lines in the maxillary portion of the palate, indicating suture fusion in this region; Stage E: Complete suture fusion. Spheno-Occipital synchondrosis staging: Stage 1: Synchondrosis completely open; Stage 2: Initial closure with superior border fused; Stage 5: Synchondrosis completely closed.

b Observed count (expected count).

c PYCD: pycnodysostosis; R: right; L: left; Zygomaticomaxillary suture.

**Table 4.** Association Between the Stages of Suture Closure and sex.

Variables		Sex (N = 18)					
Suture/synchondrosis	Stage^a^	Frequency^b^			P*	V^2^	IC
		Female	Male	Total			
Zygomaticomaxillary R	STAGE C	2 (3.1)	2 (0.9)	4	.234	.378	.112-.862
	STAGE D	2 (1.6)	0 (0.4)	2			
	STAGE E	10 (9.3)	2 (2.7)	12			
Zygomaticomaxillary L	STAGE C	2 (2.3)	1 (0.7)	3	.569	.189	.106-.780
	STAGE D	2 (2.3)	1 (0.7)	3			
	STAGE E	10 (9.3)	2 (2.7)	12			
Midpalatal suture	STAGE B	0 (1.6)	2 (0.4)	2	.095	.675	.130-1.000
	STAGE C	2 (1.6)	0 (0.4)	2			
	STAGE D	1 (0.8)	0 (0.2)	1			
	STAGE E	11 (10.1)	2 (2.9)	13			
Spheno-occipital synchondrosis	STAGE 1	0 (1.6)	2 (0.4)	2	<.001*	1.000	1.000-1.000
	STAGE 2	0 (1.6)	2 (0.4)	2			
	STAGE 5	14 (10.9)	0 (3.1)	14			

*Note*: Fisher's exact test. *p < .05.

a Zygomaticomaxillary R/L: Stage C: 2 thin lines separated by low-density spaces; Stage D: Partial suture fusion; Stage E: Multiple areas of fusion with increased parasutural bone density. Midpalatal suture stage: Stage B: A jagged, high-density line with areas showing 2 parallel lines; Stage C: 2 closely positioned, parallel, high-density lines; Stage D: 2 jagged, high-density lines in the maxillary portion of the palate, indicating suture fusion in this region; Stage E: Complete suture fusion. Spheno-occipital synchondrosis staging: Stage 1: Synchondrosis completely open; Stage 2: Initial closure with superior border fused; Stage 5: Synchondrosis completely closed.

b Observed count (expected count).

Dichotomization of suture closure stages into CLOSED versus NOT CLOSED showed a significant association with the right and left ZMS (φ = 0.707, 95% CI: 0.439-1.000, *p = .*009) and the MPS (φ = 0.620, 95% CI: 0.357-0.892, *p = .*029), but not with the SOS (φ=0.000, 95% CI: −0.478-0.500, p = 1.000). When the same CLOSED versus NOT CLOSED classification was applied, no significant associations were observed between sex and the right and left ZMS (φ=0.189, 95% CI: −0.288-0.679, *p = .*569) or MPS (φ=0.265, 95% CI: −0.254-0.791, *p = .*533). The SOS remained the only suture significantly associated with sex (φ=1.000, 95% CI: 1.000-1.000, *p < .*001).

## Discussion

Initial studies on the maturational stages of maxillary sutures focused on correlating bone maturation with chronological age, which at the time was regarded as the best clinical parameter for guiding orthodontic interventions.^20,21^ The proposed classification provides insights into the maturation stages of the MPS, ZMS, and SOS. Previous studies primarily assessed suture maturation (analyzing and comparing variables such as sex and age) and evaluated the effects of orthopedic interventions on sutures.^22–24^ Most studies to date have shown that the classification of suture maturational stages is a promising tool for predicting individual responsiveness to interventions such as rapid maxillary expansion, surgically assisted rapid maxillary expansion, or maxillary protraction in Class III patients, particularly in late adolescents and young adults.^20,22–25^ Nonetheless, there is a lack of studies evaluating facial suture maturation in patients with syndromes and/or craniofacial anomalies. Additionally, the mechanisms underlying suture closure in PYCD remain poorly understood, despite reports of craniosynostosis in affected individuals.^6^ This highlights the importance of our study, which represents the first application of this assessment method to PYCD, in a sample encompassing a broad age range (children to adults) with a matched control group.

Our findings revealed significant differences in the consolidation stages of the right and left ZMS when comparing controls and PYCD patients (*p = .*009). Furthermore, the right (*p = .*011) and left (*p = .*011) ZMS were the only sutures significantly associated with the presence of PYCD. Our control group findings align with Angelieri et al. (2017),^15^ who demonstrated the absence of fused ZMS (stages D and E) in patients under 10 years of age and a wide variability in suture stages between 10 and 15 years. In contrast, in patients with PYCD, both right and left ZMS were fused in all patients as young as 3 years old, reaffirming that pattern of ZMS closure in PYCD is distinct. Angelieri et al. (2017)^15^ also reported only the more mature side in their study, emphasizing the possibility of bilateral stage differences within the same patient. However, this was not observed in either group in our study, as all PYCD patients exhibited bilateral fusion when at the same stage. Additionally, the positive correlation between age and the right ZMS aligns with Tashayyodi et al. (2023),^26^ who applied similar CT evaluation methods in 176 patients aged 7–21 years in Iran. Using regression models, they demonstrated that age significantly impacted ZMS fusion, with each year increasing the ZMS fusion stage by 0.47 units. However, in their study, the ZMS stage in males was, on average, 0.46 units higher than in females of the same age. This contrasts with our results, where no association was found between ZMS sutures (right or left) and sex, likely due to the heterogeneity of our sample.

Midpalatal suture closure differed significantly between PYCD and control groups (*p = .*029). Although the MPS is the most extensively studied maxillary suture with this classification method, research on PYCD or comparisons with other craniofacial anomalies remains scarce. The present findings suggest that this difference may be attributed to the MPS being fully fused in PYCD patients regardless of age, whereas in controls, fusion followed an age-appropriate pattern. This understanding aligns with Ferrillo et al. (2024),^27^ who, in their study of 201 participants, demonstrated through regression analysis that MPS maturation can be influenced by several parameters, including age, sex, and vertical skeletal pattern. Shayani et al. (2023)^28^ evaluated 116 adolescents and young adults (61 females and 55 males, aged 10–25 years) using the same CBCT method and reported that stages D and E were present in 45.4% of males and 68.8% of females. Moreover, Govaerts et al. (2023),^25^ aiming to determine the age threshold for surgically assisted rapid palatal expansion in females, assessed 100 CT scans and determined the cutoff ages as 15.1 years for orthodontists and 14.8 years for oral and maxillofacial surgeons. Liu et al. (2023)^29^ also reported a strong positive correlation (*r = .*867, *p < .*001) between cervical vertebral maturation stages (CVMS) and MPS maturation stages. In contrast, a systematic review by Shayani et al. (2022)^30^ showed that while age and sex influence MPS obliteration, the correlation between these variables is weak. These results suggest that while the method for assessing MPS maturation has potential diagnostic applications, clinicians should use it with caution and conduct individualized assessments, particularly with regard to sex and age. Importantly, most previous studies have focused on typically developing patient populations and have excluded individuals with systemic conditions affecting bone metabolism, history of cleft lip or palate treatment, or other craniofacial anomalies. Therefore, our findings provide meaningful insight into PYCD, a syndrome characterized by impaired bone remodeling,^5,7^ and may also help inform the evaluation of other conditions with altered suture maturation patterns, such as cleidocranial dysplasia^31^ and osteogenesis imperfecta.^32^

The late fusion of the SOS and its role in increasing facial height and depth highlight its importance in craniofacial research. The SOS forms the cartilaginous connection between the sphenoid and basioccipital bones,^33^ a major growth site of the cranial base, typically ossifying completely by the end of the second decade of life.^34^ In this study, the SOS remained unfused only in cases and controls under 10 years of age. One major finding reported in various craniofacial syndromes is the early ossification of the SOS.^35^ In such cases, cranial base shortening is frequently accompanied by alterations in the cranial base angle (NSBa angle), defined by the Nasion-Sella and Sella-Basion lines.^36^ Consequently, midface hypoplasia may occur due to the correlation between facial height and SOS timing of ossification.^35,37^ However, our results did not demonstrate differences or associations between groups (PYCD and control) in terms of SOS fusion stages. Most studies reporting such differences relied on cephalometric radiographs and analyses^36,38^ or CT-based evaluations using simpler suture classification scales.^35^ Comparing our results with studies based on the same scale in individuals without craniofacial anomalies would not ensure full methodological alignment, although their findings were consistent with those of our control group.^39^ Furthermore, factors such as sample size, population distribution, ethnicity, and socioeconomic status may influence SOS fusion.^26^ Finally, our findings are consistent with those of Vale et al. (2020),^40^ who used the same CT methodology to assess 5 SOS consolidation stages in 125 patients aged 7–17 years, including 91 with cleft lip and palate and 34 controls, and also did not observe significant SOS differences when analyzing this craniofacial malformation.

Analyses by sex revealed a significant association with the degree of SOS consolidation (*p < .*001), consistent with Vale et al. (2020),^40^ who reported later closure in males (15 years, with and without cleft palate) compared with females (14.0 years in the case group and 13.0 years in the control group). However, Tashayyodi et al. (2023)^26^ found no sex differences in non-syndromic patients aged 7–21 years, using the same 5-point SOS fusion scale. Our findings are consistent with previous studies that used different scales and reported sex differences. Al-Gumaei et al. (2023)^41^ and Alhazmi et al. (2017)^34^ observed earlier synchondrosis fusion in females using a 4-stage scale (open, partially fused, mostly fused, and completely fused SOS). Similarly, Yang et al. (2019),^42^ employing a 6-stage scale in 262 CT images of 140 Class I skeletal patients and 122 Class III patients, also found earlier fusion (1–3 years earlier in females). The observed association between SOS fusion and sex may be explained by the faster rate of bone maturation in females, driven by hormonal factors such as estrogen.^34,43^ Additionally, the SOS serves as a cranial growth site that reflects differences in facial growth patterns between sexes. While females often exhibit early SOS closure, reflected in the NSBa angle (Nasion-Sella-Basion), and more delicate craniofacial patterns, delayed fusion in males may contribute to an elongated cranial base and, consequently, a vertically elongated facial growth.^34,39^ The chronology of bone fusion may also be partially determined by genetic factors, with bone-regulatory genes modulating fusion rates among individuals, while epigenetic factors such as nutrition and lifestyle may further influence sex-based differences. Although this association was found, it should be interpreted with caution. The small sample size and the markedly unbalanced distribution of sexes across SOS fusion stages, including categories with zero counts, may have artificially inflated the estimated effect size. Under such conditions, the observed association may reflect sample-specific characteristics rather than a true sex-related biological effect. Therefore, to avoid overinterpretation, this result should be considered exploratory and hypothesis-generating, requiring confirmation in larger and more balanced cohorts.

Lastly, a strong positive correlation between age and SOS fusion stage was observed in both PYCD and control groups (*p = .*001, *r = .*727). These findings align with those of Yang et al. (2019),^42^ who demonstrated progressive fusion stages with increasing age (*p < .*05, *r = .*824), as well as with the results of Bassed et al. (2010),^19^ who reported that fusion was well advanced by age 15 and complete by 17 years. Also, Tashayyodi et al. (2023)^26^ showed in their regression model that each additional year of age increased SOS fusion by 0.56 units. However, all earlier studies were conducted exclusively in non-syndromic individuals, suggesting potential applications for assessing craniofacial development in specific populations.^39,42^ Moreover, previous studies have demonstrated that the SOS stage determined via CBCT serves as a reliable indicator of growth maturation, showing a strong correlation with cervical vertebral maturation stages (*r = .*890), and providing external validation of this method in females (*r = .*880) and males (*r = .*890).^44^

An important aspect that should be considered in our sample is the inherent difference in skeletal pattern between groups. All patients with PYCD exhibited a Class III skeletal pattern, whereas the control group consisted exclusively of Class I individuals. The use of non-syndromic Class I individuals as controls was intended to represent typical craniofacial development and to provide a standardized reference for age- and sex-matched comparisons. Skeletal pattern is known to influence craniofacial growth direction, biomechanical loading, and suture maturation, and therefore represents a major potential confounding factor. Furthermore, the severity, craniofacial growth trajectory, and underlying biological mechanisms driving Class III malocclusion may differ substantially between syndromic and non-syndromic individuals,^45,46^ which limits direct comparison based on occlusal classification alone. As a result, observed differences in suture maturation between groups cannot be attributed solely to PYCD, as they may be partially influenced by underlying skeletal morphology. Future studies should aim to include control groups with skeletal patterns comparable to those of patients with PYCD or to evaluate suture maturation across different skeletal patterns in order to better isolate the effect of PYCD on craniofacial suture maturation.

Our findings highlight the applicability and reproducibility of CBCT-based methods for assessing maxillary suture maturation in PYCD. Nonetheless, certain limitations should be acknowledged: (I) Because PYCD is a rare disease, the sample size was inherently limited by the number of eligible patients available, which constrained our ability to generalize the findings to the broader PYCD population, (II) the sample was also unevenly distributed between sexes, limiting the ability to perform sex-based comparisons, (III) the wide age range of the sample may have introduced variability in suture maturation patterns, (IV) as this was a cross-sectional study, the exact timing of suture closure in PYCD patients could not be determined, particularly for MPS and ZMS. Despite these limitations, this study provides the first CBCT-based evaluation of facial suture maturation in PYCD and contributes important insight into craniofacial development in this rare skeletal dysplasia.

## Conclusions

This study demonstrated early fusion of the midpalatal and zygomaticomaxillary sutures in PYCD. Additional studies with larger samples are warranted to further elucidate the effects of sex and age on suture maturation, particularly the age-related fusion of the spheno-occipital synchondrosis observed in females.

## Data Availability

The datasets generated during and/or analyzed during the current study are available from the corresponding author on reasonable request.
